# Occupational cohort study of current and former workers exposed to chrysotile in mine and processing facilities in Asbest, the Russian Federation: Cohort profile of the Asbest Chrysotile Cohort study

**DOI:** 10.1371/journal.pone.0236475

**Published:** 2020-07-29

**Authors:** Joachim Schüz, Igor Bukhtiyarov, Ann Olsson, Monika Moissonnier, Evgenia Ostroumova, Eleonora Feletto, Sara J Schonfeld, Graham Byrnes, Iraklii Tskhomariia, Valerie McCormack, Kurt Straif, Sergey Kashanskiy, Tatiana Morozova, Hans Kromhout, Evgeny Kovalevskiy

**Affiliations:** 1 International Agency for Research on Cancer (IARC/WHO), Lyon, France; 2 Federal State Budgetary Scientific Institution “Izmerov Research Institute of Occupational Health” (IRIOH), Moscow, the Russian Federation; 3 I.M. Sechenov First Moscow State Medical University (Sechenov University), Moscow, the Russian Federation; 4 Cancer Council New South Wales, Cancer Research Division, Woolloomooloo, Australia; 5 Division of Cancer Epidemiology and Genetics, National Cancer Institute, National Institutes of Health, Bethesda, Maryland, United States of America; 6 Yekaterinburg Medical Research Center for Prophylaxis and Health Protection in Industrial Workers, Yekaterinburg, the Russian Federation; 7 Institute for Risk Assessment Sciences, Utrecht University, Utrecht, The Netherlands; University of Cincinnati College of Medicine, UNITED STATES

## Abstract

A historical cohort study in workers occupationally exposed to chrysotile was set up in the town of Asbest, the Russian Federation, to study their cause-specific mortality, with a focus on cancer. Chrysotile has different chemical and physical properties compared with other asbestos fibres; therefore it is important to conduct studies specifically of chrysotile and in different geographical regions to improve the knowledge about its carcinogenicity. Setting was the town of Asbest, Sverdlovsk oblast, the Russian Federation. Participants were all current and former employees with at least one year of employment between 1/1/1975 and 31/12/2010 in the mine, enrichment factories, auto-transport and external rail transportation departments, the central laboratory, and the explosives unit of the company. Of the 35,837 cohort members, 12,729 (35.5%) had died (2,373 of them of cancer, including 10 of mesothelioma), 18,799 (52.5%) were known to be alive at the end of the observation period (2015), and 4,309 (12.0%) were censored before the end of 2015. Mean follow-up duration was 21.7 years in men and 25.9 years in women. The mean age at death was 59.4 years in men and 66.5 years in women. This is the largest occupational cohort of chrysotile workers to date, and the only one with a large proportion of exposed female workers.

## Introduction

All commercially exploited forms of asbestos (amosite, anthophyllite, chrysotile, and crocidolite), and varieties not widely used in industry (as tremolite and actinolite) are known to cause cancer in humans, with sufficient evidence that asbestos causes cancers of the lung, larynx, and ovary as well as mesothelioma [1]. In addition, positive associations have been observed between exposure to asbestos and cancers of the stomach, pharynx, colon, and rectum [2]. In 2007, the Sixtieth World Health Assembly requested member states of the World Health Organization (WHO) to conduct global campaigns for the elimination of asbestos-related diseases [3, 4]. Following this request, the Ministry of Health and Social Development of the Russian Federation issued an order to develop a National Program for Elimination of Asbestos-Related Diseases including an epidemiologic investigation of the association between occupational and non-occupational exposure to chrysotile asbestos and cancer risk and mortality [5].

Chrysotile is the most commonly used form of asbestos worldwide and the only one that is still commercially mined. As with most identified carcinogens, there remain unanswered questions on the nature of cancer risks. The rationale for initiating this occupational cohort study of current and former workers exposed to chrysotile in mines and enrichment facilities in Asbest, the Russian Federation, has previously been published [6]. In short, chrysotile has different chemical and physical properties compared with other asbestos fibres [7]. Therefore, it remains important to conduct studies specifically of chrysotile and in different geographical locations to expand the knowledge about its carcinogenicity, as distinct from that of amphiboles or mixtures of chrysotile and amphiboles [6–8]. This is particularly important given the prolonged disease burden due to chrysotile, which arises for two reasons. First, asbestos-related cancers typically arise several decades after the first exposure, as demonstrated in countries that now have the highest mesothelioma mortality rates worldwide, all of which have long banned the use of asbestos (e.g., Australia, Germany) [9–11]. Second, even in the absence of active mining and use of chrysotile, exposure to chrysotile would be expected to continue worldwide due to its persistence in the environment (e.g., the presence of chrysotile in the Earth’s crust) and ongoing exposures such as those arising from repair or removal work (e.g., related to maintenance or demolition of materials containing chrysotile).

The Asbest Chrysotile Cohort was set up to obtain more precise quantification of the site-specific cancer risks. In particular, for lung cancer and mesothelioma, more precise quantification of the magnitude, exposure–response relationship, and timing of the cancer risks are needed. Further evidence of the chrysotile-associated risks of cancers of the ovary and larynx, recently classified as asbestos-related cancers, will be assessed. In addition, the cohort will examine cancers for which there is some, but inconclusive, evidence of an association with chrysotile, such as cancers of the pharynx, stomach, colon, and rectum [12]. This report is a thorough cohort description with information on the study setting, cohort enrolment, exposure assessment, follow up for vital status and cause of death, and the planned risk analyses, as well as a discussion of strengths and limitations of the study. Statistical methods used are calculations of frequencies (presented as tables or bar charts) and a boxplot showing exposure distributions.

## Cohort description

### Study setting

**Fig 1:**
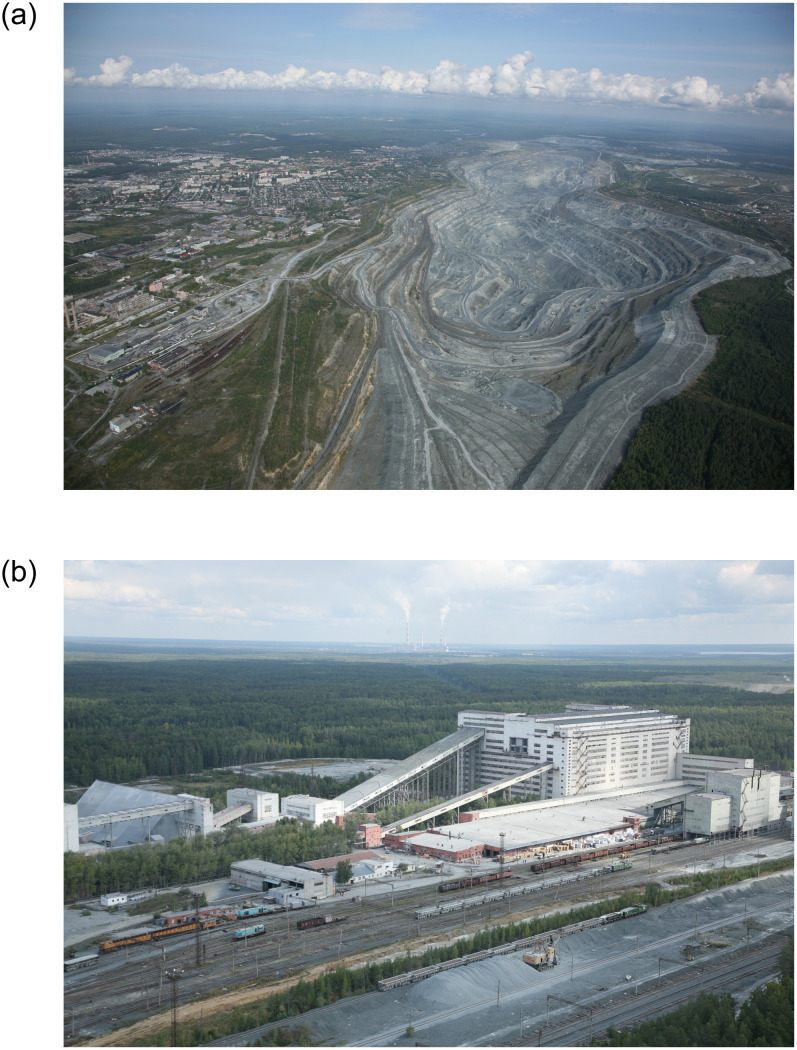
**A**. Open-pit mine in Asbest, Sverdlovsk oblast, the Russian Federation (photo provided by JSC Uralasbest). **B**. Enrichment factory #6 of JSC Uralasbest, Asbest, Sverdlovsk oblast, the Russian Federation (photo provided by JSC Uralasbest).

In Asbest, the Joint Stock Company (JSC) Uralasbest operates the world’s largest open-pit chrysotile mine (Bazhenovskoye deposit of chrysotile asbestos) and its enrichment factories [13]. This site currently produces approximately 20% of the world’s chrysotile and has been in operation for more than 120 years. The open-pit mine has increased in size over time and now covers an area of 12 km^2^ and reaches a depth of 325 meters, see Fig 1a, and is located in Sverdlovsk oblast. An “oblast” in the Russian Federation is a federal administrative division, similar to a province, and Sverdlovsk oblast is spread over the slopes of the North and Middle Urals and the Western Siberian plain, with about 4.3 million inhabitants. Chrysotile fibres properties in general vary widely, as described by numerous authors [14]. Chrysotile mined in Asbest covers the full range of strengths and lengths for uses ranging from slate, insulation, to textiles, among others [15]. Geological investigations by JSC Uralasbest and local scientific institutions suggest that in the Asbest chrysotile deposit other asbestos types are absent or, at most, minimal; some manifestations of tremolite, actinolite, and other asbestiform fibres were discovered far away from the ore field of the chrysotile mining area [16, 17]. A study of fibres in lung tissue found small amounts of amphiboles in samples from persons both with occupational and without occupational exposure, according to the authors indicating that the major source of amphiboles (tremolite/actinolite) may be impurities of ore, wastes, rocks, and soil, whereas finished chrysotile products contributed to a lesser extent [18].

Chrysotile is mined through a process of drilling and blasting, with the excavated rocks transported via trucks at the lower and middle levels of the pit and then via rail to an asbestos-enrichment processing mill a few kilometres away [19], see Fig 1b. Since the late 1800s, there have been 7 asbestos-enrichment processing mills (called factories) operated by JSC Uralasbest, each of which conducted the same chrysotile-enrichment process, but with varying technology and machinery used. Over time, the degree of mechanization has advanced and technologies have improved, permitting the enrichment of ore with lower chrysotile content and resulting in reduced exposures to airborne dust and fibres in the work environment [20, 21]. All factories used the dry milling technique, which consists of repeated cycles of crushing the raw ore, drying, and then screening through vibrating screeners so that chrysotile fibres rise to the surface and are removed by vacuum suction. The majority of production today results in 50 kg packages of chrysotile, graded for use in pipes, slate, textiles, and fillers.

The epidemiological study design of the Asbest Chrysotile Cohort is a historical cohort study of current and former employees of JSC Uralasbest. The data are based on company records, and via record linkage followed up for mortality obtained from official death certificates of Sverdlovsk oblast. Therefore, cohort enrolment and follow-up did not require individual contact with or consent from study participants. The study was approved by the International Agency for Research on Cancer (IARC/WHO) Ethics Committee (IEC No. 12–22, September 2012). The IEC and an independent Scientific Advisory Board (see Acknowledgements) monitor the progress of the study on an annual basis.

### Cohort enrolment

The cohort includes all current and former employees with at least one cumulative year of employment between 1/01/1975 and 31/12/2010 of the following JSC Uralasbest enterprises: the mine, all factories (Oktyabrskaya (factory 0) and factories 1 to 6), auto-transport and external rail transportation departments, the central laboratory, and the explosives unit at the company. Eligible cohort members thus included both workers who were already employed in 1975 and workers who were newly hired in 1975 or afterwards. Employees with less than one cumulative year of employment after 1975 were not considered eligible for inclusion (irrespective of when they started), because this group would comprise large numbers of short-term staff (mainly interns and trainees) for which no data on their subsequent occupational history would be available. Note that this eligibility criterion could be applied only after the complete work history had been extracted from the archives (in earlier years, the mine and each factory had their own independent archives) and the cumulative employment duration had been calculated. Employment in or after 1975 was defined for inclusion of cohort members, because for earlier time periods pilot work indicated that follow-up would probably be incomplete and of lower quality. Employment in or before 2010 was defined for inclusion in the cohort, because for those employed after 2010 only, the follow-up time was considered to be too short to be informative with regard to cancer mortality, because of the long latency period between first exposure and disease. The chosen enterprises were those where exposure to chrysotile was likely and measurements of airborne dust were performed systematically. Until the mid-1990s, within each of the enterprises included in the cohort, in addition to workers directly involved in chrysotile production, enterprise employees—and thus cohort members—included accountants, social workers, canteen staff, kindergarten personnel, and other support personnel. Therefore, each enterprise included workers with no, low, and potentially high occupational exposure to chrysotile, generating a cohort with informative internal exposure contrasts.

Enrolment of the cohort involved the following steps to ensure cohort completeness according to the eligibility criteria outlined above. First, current employees in 2010 were identified from the then-newly created central electronic database of JSC Uralasbest employees of all the company’s enterprises, including their work history information at JSC Uralasbest.

Second, from the paper archives of the various enterprises, a study data entry team obtained the work cards (worker registration cards) for employment periods ending between 1975 and 2009. Worker registration cards are indexed by the end date of employment in an enterprise, and consequently workers have multiple worker registration cards when they change enterprises within the company. In this and subsequent stages, the data entry team checked each full name, sex, and date of birth on the work card against the study database to determine whether a record already existed for a given worker. If so, the work history information on the work card was checked against the information in the evolving study database (from the electronic records and/or other work cards) and any information not previously captured was added to a worker’s record. A new record was created for any worker identified in the work cards for which there was no match in the database.

Third, for some workers complete work histories could be ascertained from personal workbooks (a personalized paper booklet with information of the entire work history of the worker). For active workers and living retired workers residing in the town of Asbest who agreed to provide a copy of their personal workbook, this information was also entered into the study database.

In the next step, the information from archived work cards from the period before 1975 (back to 1950) was extracted to complete the work history of all workers with at least one record in the study database (current worker, workbook, or at least one work card from 1975 or later). Hence, for each cohort member, the full work history while working at JSC Uralasbest was available. This applied to all cohort members, i.e., existing workers in 1975 and newly hired workers in 1975 or later, those working for several different enterprises within the company, and those with gap years in their full employment history.

Several extensive procedures were applied to detect duplicates and to link database entries belonging to the same cohort member; such duplicates could have been due to differences in the spelling of names, name changes (e.g., due to marriage, divorce, or other reasons) during the employment period, or typing errors in any of the personal identifying data. First, a software package (RecLink 3.3, written by Andy Cook) was used to find matching entries missed during data entry, enabling manual checking of potential matches of similar but not identical spelling of names or birth dates. Multiple runs were applied, and manual checking was done to complement the automatic searches. Second, after obtaining information on changes to cohort members’ names from the Civil Act Registration Office (ZAGS; abbreviation based on the Russian name) of Sverdlovsk oblast, searches for matches using multiple names were conducted and potential duplicates were manually checked.

Salary books were also available and listed all workers, by enterprise, who received a salary in a given year. A check against a random sample of salary book records was carried out to ensure completeness of the cohort over time. In a check of 500 individuals from the salary books, all 500 people from the salary books were found in the cohort study database.

**Fig 2:**
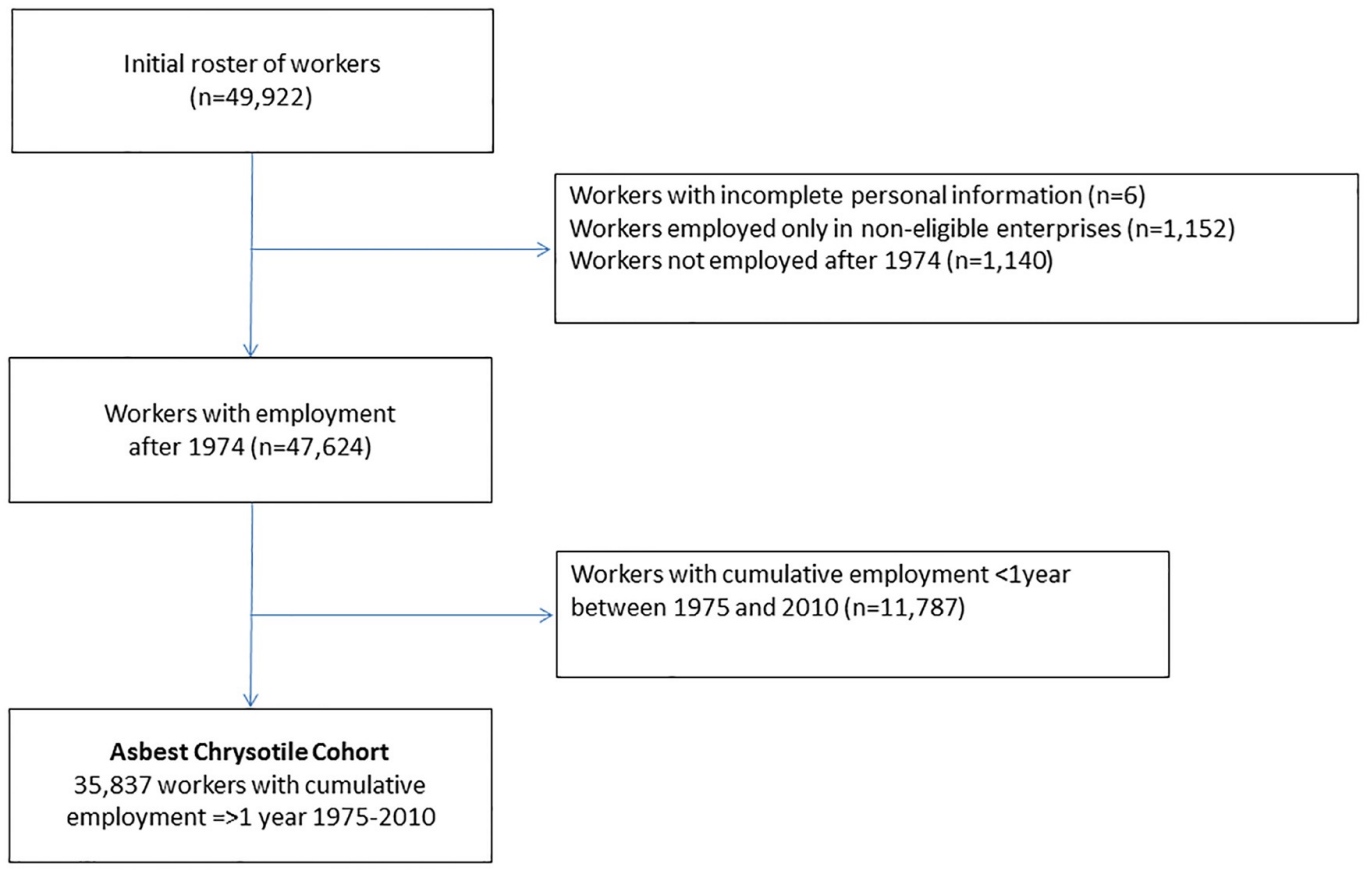
Flow chart of enrolment in the Asbest Chrysotile Cohort study.

**Fig 3:**
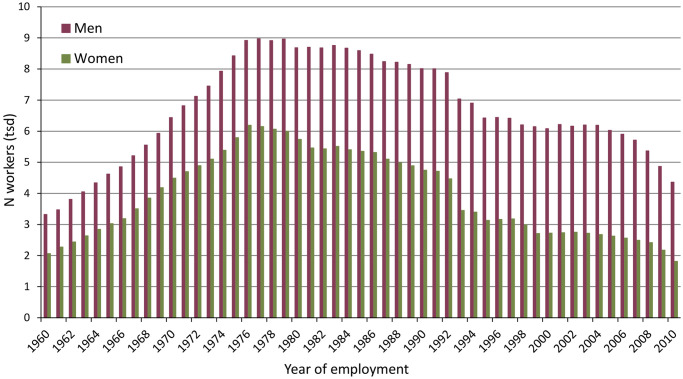
Number of active workers in thousands (tsd) by year in the Asbest Chrysotile Cohort study.

Fig 2 is a flow diagram illustrating the enrolment of the cohort. As noted above, the eligibility criterion of having worked for at least one cumulative year could be checked only after the work history information from all worker registration cards had been entered. Thus, the initial roster of workers included more than 49,000 subjects, of which 35,837 define the Asbest Chrysotile Cohort. Table 1 shows the composition of this final cohort. Female workers constitute a substantial portion of the cohort (37%). The majority of workers in the cohort started working at JSC Uralasbest before the age of 25 years. Due to the high mining activity in the 1970s and the study design with the start of enrolment of existing workers in 1975, the greatest proportion (24%) of cohort members was first employed in the 1970s. The median duration of employment at JSC Uralasbest for the cohort members was 11 years (mean, 14.4 years); 53% of the cohort had worked there for more than 10 years and 31% for more than 20 years. More men than women were employed in the mine (76% of mine workers were men). Fig 3 shows the number of active workers by year, with the respective numbers of men and women. Among the enrolled cohort, due to the study design, 1975 and 1976 were the years with the largest number of cohort members employed at JSC Uralasbest. There were fewer workers in the 1990s because of the economic situation following the dissolution of the Soviet Union. The mean follow-up time for cohort members was 21.7 years in men and 25.9 years in women.

**Table 1:** Employment-related characteristics of the workers in the Asbest Chrysotile Cohort study.

Characteristic	Men	Women	Total
	N (%)	N (%)	N (%)
Total*	22,463 (63)	13,374 (37)	35,837 (100)
Age at first year of employment at JSC Uralasbest (years)			
Less than 20	8,642 (38)	4,748 (35)	13,390 (37)
20–24	7,064 (31)	3,574 (27)	10,638 (30)
25–29	2,808 (13)	1,954 (15)	4,762 (13)
30–34	1,417 (6)	1,161 (9)	2,578 (7)
35–39	925 (4)	856 (6)	1,781 (5)
40 or older	1,607 (7)	1,081 (8)	2,688 (8)
Calendar year of first year of employment at JSC Uralasbest			
Before 1960	3,613 (16)	2,245 (17)	5,858 (16)
1960–1969	3,413 (15)	2,508 (19)	5,921 (17)
1970–1979	5,349 (24)	3,406 (25)	8,755 (24)
1980–1989	4,311 (19)	2,926 (22)	7,237 (20)
1990–1999	3,484 (16)	1,508 (11)	4,992 (14)
2000 or later	2,293 (10)	781 (6)	3074 (9)
Duration of work (years)			
1–4	7,130 (32)	3,467 (26)	10,597 (30)
5–9	3,809 (17)	2,410 (18)	6,219 (17)
10–14	2,853 (13)	1,921 (14)	4,774 (13)
15–19	1,964 (9)	1,441 (11)	3,405 (10)
20–24	1,688 (8)	1,413 (11)	3,101 (9)
25–29	1,665 (7)	1,175 (9)	2,840 (8)
30 or more	3,354 (15)	1,547 (12)	4,901 (14)
Main employment setting			
Mine/External rail	13,429 (60)	4,352 (33)	17,781 (50)
Enrichment factory/Central lab	3,974 (18)	6,206 (46)	10,180 (28)
Both	5,060 (23)	2,816 (21)	7,876 (22)
Age at last year of employment at JSC Uralasbest (years)**			
Less than 30	5,469 (24)	2,971 (22)	8,440 (24)
30–39	4,383 (20)	2,494 (19)	6,877 (19)
40–49	4,044 (18)	3,289 (25)	7,333 (20)
50 or older	8,567 (38)	4,620 (34)	13,187 (37)

* Row percentage for men and women for Total, otherwise column percentage

** Irrespective of reason for leaving (e.g., retirement, dismissal, death).

### Exposure assignment

For each eligible cohort member, the following information was extracted from the company’s records: last name, first name, patronymic name, changes in last name (if applicable and recorded), sex, birth date, and place of birth (for data linkage) and start date, end date, enterprise, work unit within enterprise, and job title for each job held (for exposure assessment). For job titles, because job designations changed over time, comprehensive dictionaries were created so that comparable activities had the same notation in the study database.

For exposure assessment, the occupational history was linked with a company-specific job–exposure matrix constructed from a database of more than 90,000 measurements of airborne dust concentrations taken until 2002 (the individual steps are described below). Dust concentrations were available from regular and systematic sampling across workplaces in the factories (since 1951) and the mine (since 1964) conducted mainly by the company’s central laboratory [20]. Measurements were taken at stationary sampling points in representative areas where workers conducted their work (work area). Steps for the exposure assessment are detailed in a separate manuscript in preparation, but in brief the following steps were undertaken. For each job, an annual average dust concentration was estimated, which was derived from measurements taken at the measurement points applicable to that job (note that jobs with mobility of workers may have involved several measurement points). In the mine, the annual average was weighted by season, by calculating first averages for the winter and the summer period and weighing them 2:1 to reflect the relative length of winter versus summer in this area [21]. Estimated annual average concentrations were linked to each cohort member based on the job performed in each calendar year and adjusted for the proportion of the year that the worker had actually worked. Thus, for each individual, cumulative exposure to dust was estimated for their entire occupational history at JSC Uralasbest. For years in which measurements were not available because they were either missing or not collected systematically using the same measurement method, annual dust concentrations were extrapolated using linear mixed models. Actual measurements of dust concentrations covered 87% of the working years in the factories for which exposure had to be estimated and 76% in the mine (person-time accumulated in eligible jobs).

Exposure to asbestos fibres was estimated using dust-to-fibre conversion factors derived from three series of parallel measurements of dust and fibre concentrations conducted in 1995, 2007, and 2013–2014; these conversion factors were used to assign modelled fibre concentrations to each annual average dust concentration [21]. Estimated fibre concentrations were then assigned to each cohort member by job and by calendar year. This resulted in cumulative fibre years of exposure as another exposure index.

In conclusion, dust exposure is based on systematic detailed measurements over more than 60 years, while fibre exposure is mainly modelled using dust-to-fibre conversion factors derived from three series of parallel measurements. The latter were however from more recent periods, i.e. not before 1995, so that particularly for the high exposure levels occurring in the first half of observation time of the study no data for direct comparison exists and modelling assumptions had to be made. Cumulative dust exposure will therefore be the primary exposure metric in the risk analysis, whereas cumulative fibre years of exposure will be a second exposure metric.

For both the dust exposure and the fibre exposure metrics, an extra source of information was available. The JSC Uralasbest Occupational Safety Department assigned to each job title by time, unit, and factory a percentage called “percentage of working time in exposed areas”, which varies from 0% to 100% and reflects the potential exposure during a working day (actual amount of time during an average working day that the worker spends at a certain workplace, mainly for jobs where working time is spent outside exposed areas as part of the job activities); this is a mandatory assessment according to the legislation in the Russian Federation. However, the percentage of working time in exposed areas is an indicator of all potential hazardous exposures, and although exposure to dust can be assumed to be among the major ones, it also includes exposures such as to noise. Both cumulative dust exposure and cumulative fibre years of exposure will be adjusted by this percentage and the resulting adjusted metrics applied in sensitivity analyses.

**Fig 4:**
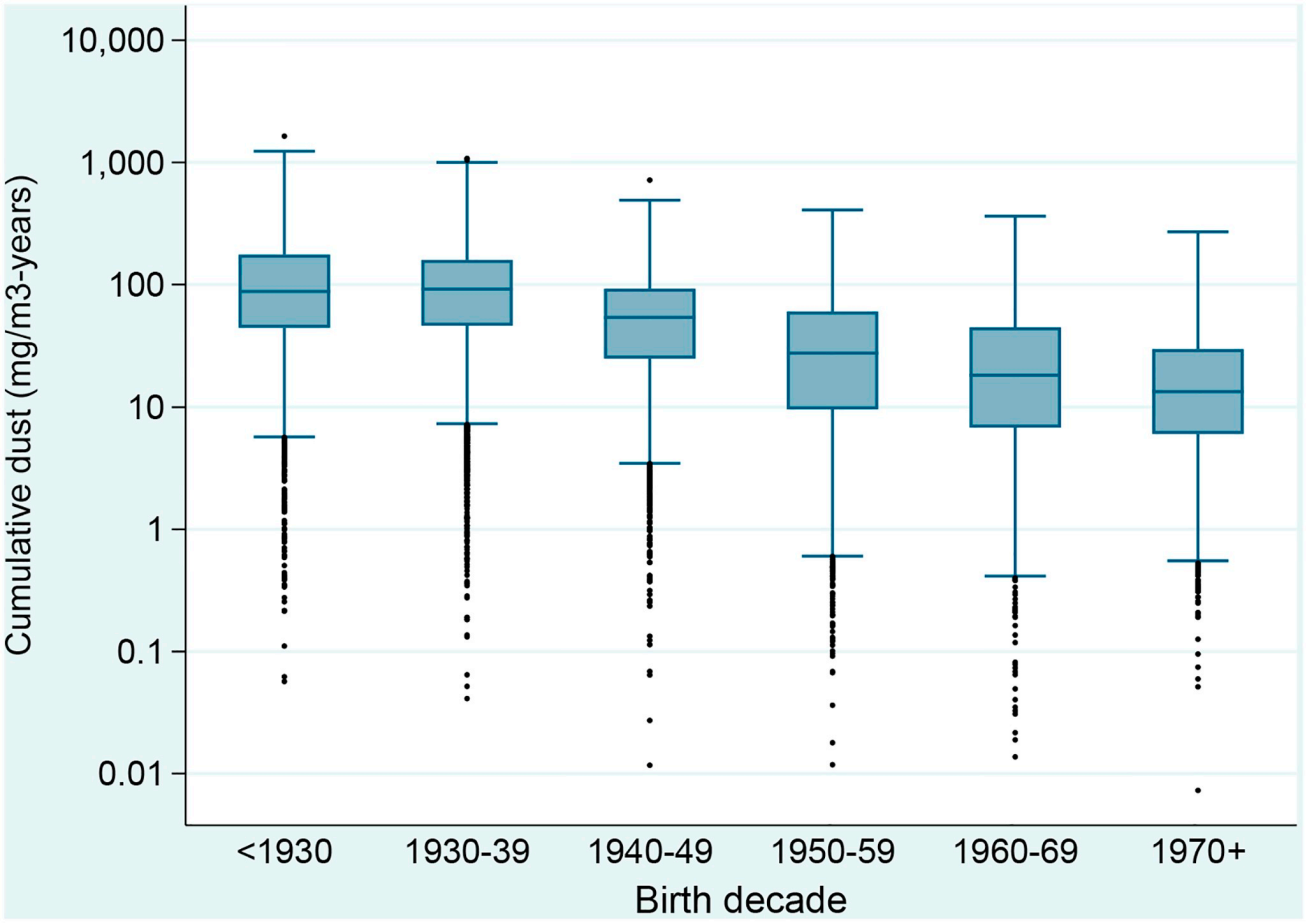
Box plot of cumulative exposure to dust (mg/m^3^-years) of workers in the Asbest Chrysotile Cohort study, by birth decade. The dark line in the middle of the boxes represents the median, the bottom of the box indicates the 25^th^ percentile, and the top of the box represents the 75^th^ percentile.

Fig 4 shows box plots of the cumulative exposure to dust by the birth decade of workers, illustrating decreasing cumulative exposure over time but also showing marked exposure contrasts within all birth decades.

### Vital status follow-up

Vital status is essential for the censoring of risk time. With the aim of achieving the highest completeness in follow-up, multiple information sources were used. December 31, 2015, was set as the end date for follow-up, because soon thereafter the process of obtaining permissions for data linkage with the Civil Act Registration Office (ZAGS) and other registries was initiated, and the end date had to be specified in the respective contracts. First, company records were used to identify current workers. The Veterans Council’s records for retired workers of JSC Uralasbest residing in Asbest was an additional resource to identify living cohort members. Second, as a major source for identifying deceased cohort members, ZAGS provided the date and cause of death of those deceased in Sverdlovsk oblast from the start of the follow-up on1/1/1976 until the end of 2015. In addition to ZAGS, data were retrieved from the more recently established cause-of-death registry of the Medical Information and Analytical Center (MIAC) of Sverdlovsk oblast, which also receives information from the medical death certificates and performs the information extraction and cause of death coding independently of ZAGS. MIAC cause-of-death data were available only from 1990 onwards and were mainly used for checking the completeness of the ZAGS-based mortality follow-up (and, in fact, the completeness was confirmed) [22]. In addition, data from the Pension Fund and the Federal Migration Service of the Russian Federation were used to ascertain the vital status of the remaining cohort members not found through any other record linkages. Specifically, the Pension Fund data were used to identify those receiving a pension in 2015 or for whom their employers (in addition to JSC Uralasbest) made contributions to their pensions in the Pension Fund, confirming that those cohort members were still alive and resided either within the oblast or had left it. The Federal Migration Service data were used to identify those who had migrated away from the oblast. However, for some cohort members, the date of migration was not recorded, and hence they had to be censored at the last date when they were known to be alive and resided in Sverdlovsk oblast (from employment records or any available civil act certificate, such as marriage or divorce).

Record linkages were performed by the respective authorities based on personal identifiers. In ZAGS, in the first step, deterministic record linkage using all names and date of birth was done, but later some manual searches of cohort members were done, in particular among those who had potentially died in the early years of follow-up, to reduce the number of missed matches due to errors in the personal identifiers. MIAC has a stochastic record linkage that is implemented by using various combinations of the personal identifiers, including using initials instead of first names and only parts of the birth date instead of only the complete birth date; matches above a certain matching likelihood threshold were manually checked to determine whether they were true matches [22]. Because of their massive database size, the Pension Fund and the Federal Migration Service could only perform deterministic record linkages based on all personal identifiers; otherwise, the numbers of false-positive matches would have been overwhelming.

Overall, of the 35,837 cohort members, 12,729 (35.5%) are known to have died and, 18,799 (52.5%) were known to be alive at the end of the follow-up period (2015). The remaining 4,137 (11.5%) were censored before the end of 2015. Only 172 (0.5%) cohort members had no successful match in record linkage with any of the above-mentioned sources, and those were also censored before the end of the follow-up, at the last known year of their employment at JSC Uralasbest. There were no differences by sex or by birth cohort between those followed up until 2015 and those who were censored before the end of 2015.

### Causes of death

For each cohort member who died within Sverdlovsk oblast, the cause of death was derived from the ZAGS electronic death certificates’ database, based on deterministic record linkage complemented by manual searches of cohort members. ZAGS provided individual’s causes of death as original text information, as causes of death were only available coded according to the principles of the International Statistical Classification of Diseases and Related Health Problems (ICD) if done by the medical practitioner filling in the death certificate. Because this study spanned such a long time period (from 1975 to 2015), the cause-of-death coding practices and classifications officially used in the Soviet Union/Russian Federation changed several times (from ICD-8 to ICD-10 revision) [22]. Hence, it was decided to use the original text information of all listed causes of death (immediate cause, underlying cause, additional causes if applicable) from the death certificates of deceased cohort members, and to manually code the underlying cause of death according to the official coding instruction of ICD 10th revision. The coding was done by a Russian speaking medical doctor with professional experience in medical classifications working at IARC/WHO. From the stochastic record linkage with MIAC, as described above, the causes of death were obtained already coded for 1990–2001 using MIAC-specific non-ICD-based nomenclature, and from 2002 onwards using ICD-10, but the original cause-of-death text was not recorded and stored at the MIAC death registry. Therefore, only ICD-10 coded MIAC data from 2002 onwards were used to validate the manual ICD-10 coding done at IARC/WHO based on the ZAGS data, and the results of the comparison and more details on death data retrieval from ZAGS and from MIAC are published elsewhere [22]. The main figure confirming the high quality of coding is the agreement of 96.4% for 1,009 cancer deaths coded independently by IARC/WHO and by MIAC.

**Fig 5:**
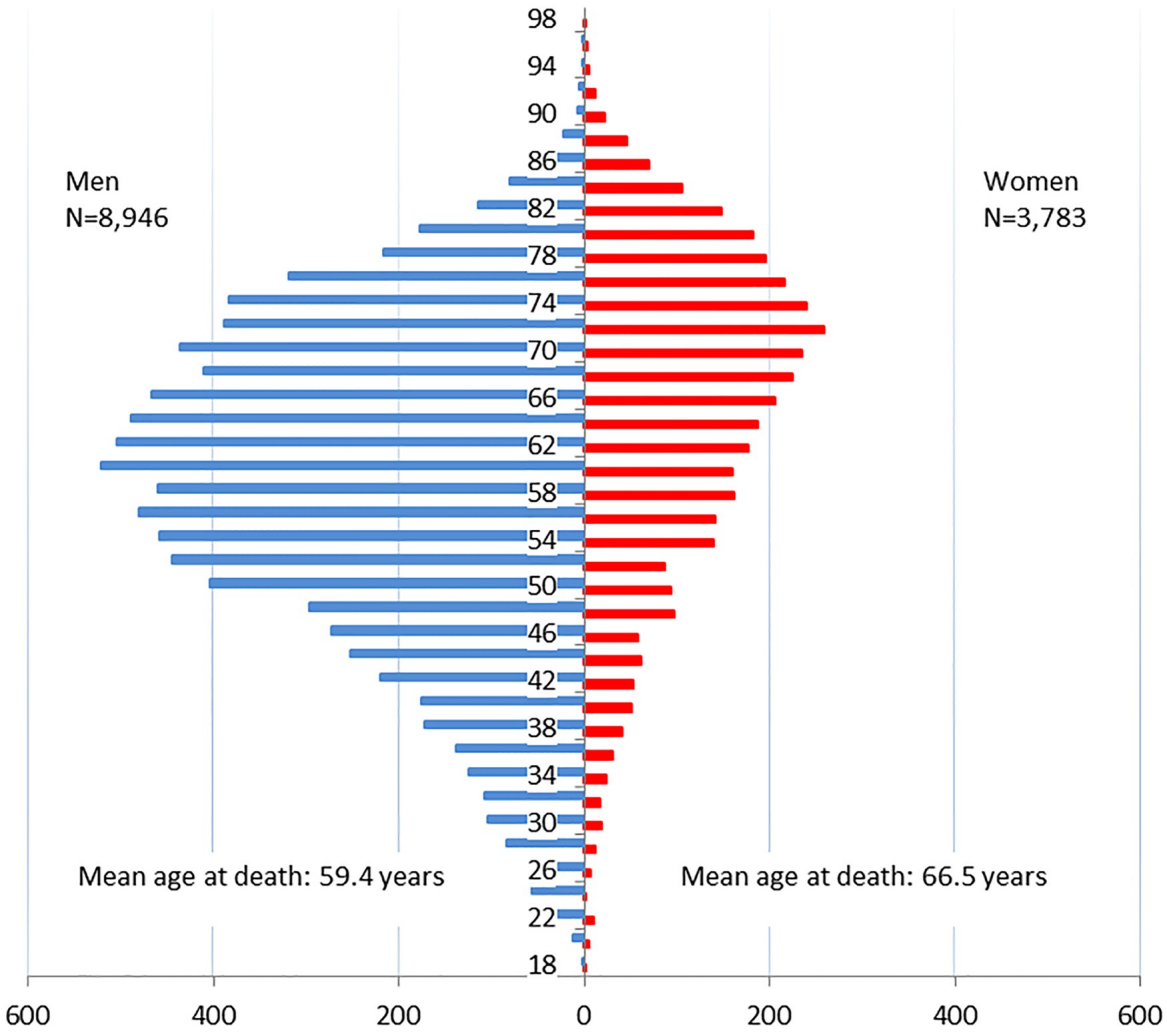
“Age at death” distribution of 12,729 deceased male and female cohort members (1976–2015) (left in blue = men, right in red = women).

In short, we consider that the ascertainment of causes of death for the cohort members who died in Sverdlovsk oblast in 1976–2015 successfully tracked at vital status follow-up was virtually complete. Of the total of 12,729 deaths observed from all causes, the most frequent cause of death was circulatory system diseases, followed by external causes and cancer in men, and by cancer and external causes in women, see Table 2. Cancer mortality was dominated by lung cancer in men and breast cancer in women, see Table 3. Ten workers had died of mesothelioma, of which two were women, and their ages at death ranged from 48 to 76 years. The overall age-at-death distribution by sex is shown in Fig 5; the mean age at death was 59.4 years in men and 66.5 years in women.

**Table 2:** Main causes of death in the Asbest Chrysotile Cohort study, 1976–2015.

Cause of death (ICD-10)	Number of deaths (%)		
	Men	Women	Total
All causes	8,946 (100)	3,783 (100)	12,729 (100)
Neoplasms (C00-D48)	1,682 (18.8)	720 (19.0)	2,402 (18.9)
Circulatory system diseases (I00-I99)	3,897 (43.6)	2,017 (53.3)	5,914 (46.5)
Respiratory system diseases (J00-J99)	353 (3.9)	87 (2.3)	440 (3.5)
Digestive system diseases (K00-K93)	471 (5.3)	220 (5.8)	691 (5.4)
External causes (S00-T98)	1,794 (20.1)	327 (8.6)	2,121 (16.7)
Symptoms, not elsewhere class. (R00-R99)	222 (2.5)	140 (3.7)	362 (2.8)
Other causes	372 (4.2)	205 (5.4)	577 (4.5)
Missing causes	155 (1.7)	67 (1.8)	222 (1.7)

**Table 3:** Cancer deaths in the Asbest Chrysotile Cohort study, 1976–2015.

Cause of death (ICD-10)	Number of deaths (%)		
	Men	Women	Total
All cancers (C00-C97)	1,662 (100)	711 (100)	2,373 (100)
Site-specific cancers:			
Pharynx (C10-C11, C13-C14)	12 (0.7)	1 (0.1)	13 (0.5)
Stomach (C16)	199 (12.0)	95 (13.4)	294 (12.4)
Colon and rectum (C18-C21)	151 (9.1)	99 (13.9)	250 (10.5)
Liver (C22)	56 (3.4)	16 (2.3)	72 (3.0)
Larynx (C32)	51 (3.1)	2 (0.3)	53 (2.2)
Lung (C33-C34)	622 (37.4)	57 (8.0)	679 (28.6)
Breast (C50)	1 (0.1)	129 (18.1)	130 (5.5)
Cervix (C53)	-	21 (3.0)	21 (0.9)
Ovary (C56)	-	50 (7.0)	50 (2.1)
Prostate (C61)	64 (3.9)	-	64 (2.7)
Kidney (C64)	72 (4.3)	14 (2.0)	86 (3.6)
Bladder (C67)	38 (2.3)	5 (0.7)	43 (1.8)
Ill defined, second., unspecific (C76-C80)	63 (3.8)	23 (3.2)	86 (3.6)
Haematological (C81-C96)	64 (3.9)	42 (5.9)	106 (4.5)
Mesothelioma (C38.4, C45)	8 (0.5)	2 (0.3)	10 (0.4)
Other cancer sites	261 (15.7)	155 (21.8)	416 (17.5)

### Covariates

Individual data on smoking in the cohort were not available. Although this is common in occupational cohort studies based on archive and registry data, smoking remains an important potential confounder in investigating tobacco-related cancers, especially if smoking varies by job. To examine smoking patterns by sex, calendar time, and job, a survey independent of the cohort study was conducted among active workers and living pensioners of JSC Uralasbest residing in Asbest; more details are published elsewhere [23]. In brief, among 2,096 male and 897 female active workers, the prevalence of ever-smoking was 68.7% and 19.5%, respectively, and among 1,596 male and 2,836 female pensioners, the prevalence of ever-smoking was 63.2% and 5.9%, respectively. No association between smoking status and cumulative dust exposure was seen in male workers after adjusting for birth decade. In female workers, the prevalence of smoking was higher in exposed workers compared with a reference group of female workers with no professional exposure, but the prevalence did not vary across the categories of cumulative dust exposure.

### Statistical analyses

A data analysis plan was developed by the study team in collaboration with the Scientific Advisory Board and posted on the study website: https://asbest-study.iarc.fr in December 2019. In short, all-cause mortality and site-specific mortality from cancer of the trachea and lung (ICD-10 codes C33-C34), mesothelioma (C45, including and excluding “malignant neoplasm of pleura” C38.4), larynx (C32), pharynx (C10-C11, C13-C14), stomach (C16), colon and rectum including anorectal (C18-C21), ovary (C56), and “all other cancers” (ICD-10: C00-C97 excluding those listed above) will be analysed. The association with all-cause mortality and lung cancer will be investigated using Poisson regression models with five exposure categories, namely workers with no professional dust exposure, 1^st^ and 2^nd^ tertiles of exposed workers, and the 3^rd^ tertile of exposed workers split into the 75%-90% quantile and the 10% with highest exposure. The tertiles will be assessed using cumulative exposure in all exposed study cohort members, male and female. The association with cancers other than lung will be investigated using Poisson regression models with three exposure categories (due to smaller numbers), namely workers with no professional dust exposure, and workers exposed below and above the median of cumulative dust exposure. All analyses will apply a 5-year lag of cumulative dust exposure from the end of follow-up, and will be stratified by sex. Adjustments will be made by age and age-squared in 5-year categories, for birth decade to account for potential confounding of tobacco smoking, and for calendar period. Since it has been observed that smoking patterns are different in women without professional chrysotile exposure, care will be taken in comparing that group with the other exposure categories. For endpoints where numbers allow, the exposure-response function will be further explored using spline techniques allowing also for non-linear shapes. In sensitivity analyses, the effect of lag time will be explored by using longer lag times of 10 and 20 years applied to lung cancer and other cancers where the number of cases is sufficient.

The cohort data files (cohort members’ demographic data, vital status, cause of death for deceased) and the dust exposure data were preserved by IARC/WHO on 25/10/2019 producing the following SHA256 Hash code:

2D55CF6BE6C383671AD6733EE04DB4F2FE446A40B2410884C3837B6B2377EAFA

The SHA256 hash is a one-way function that produces a fixed size text for any size of source data. It is not an encryption therefore the hash cannot be decrypted back to the original data. However, any future SHA256 hash calculation of the file will result in the same hash value only if the data remains unmodified.

Raw data cannot be made publicly available according to the data confidentiality legislation of the Russian Federation. Anonymisation of records would not suffice to exclude the possibility of re-identification of individual workers due to the detailed occupational history collected in the cohort. Aggregate original data will be published on the website of the International Agency for Research on Cancer (IARC/WHO) at http://asbest-study.iarc.fr at the time of publication of the risk analysis.

### Findings to date

To date, time trends in the measured dust concentrations and results from the parallel measurements of dust and fibre concentrations have been published [20, 21], briefly described below.

Dust concentrations from measurements across workplaces in the factories and the mine were analysed for time trends [20]. Based on 89,290 dust concentration measurements between 1951 and 2001 in six factories operating in different but partially overlapping time periods, annual mean dust concentrations in the early years were as high as 192 mg m^-3^ while they were between 5 and 2.8 mg m^-3^ in later years in the factory still in operation today. Overall trends in the factories varied across decades, with the steepest annual declines observed before 1960 (−21.5% in factory 2 and −17.4% in factory 3), more moderate declines in the 1960s and 1970s (ranging from −10% in factory 2 in the 1960s to −0.3% in factory 4 in the 1970s), and little change thereafter. Based on 1,544 dust concentration measurements in the mine since 1964, the annual mean dust concentration increased from 2.7 mg m^−3^ in 1964 to 7.8 mg m^−3^ in 1966 and then decreased to 4 mg m^−3^ in 1969. In the 1970s and early 1980s annual mine dust concentrations ranged from 2.5 to 4.7 mg m^−3^ with no clear pattern of increasing or decreasing levels, but there were overall decreases from the mid-1980s to the end of the 1990s and early 2000s with annual mean dust concentrations in these later years ranging from 1.8 to 2.2 mg m^−3^. In short, in the mine, dust concentrations increased in the 1960s (+9.7% per year), decreased in the 1990s (−5.8% per year), and were stable in between.

Parallel measurements of dust concentrations and fibre concentrations were taken in 1995, in 2007, and for the purpose of the Asbest Chrysotile Cohort study in 2013–2014, both in operating factories and in the mine [21]. From the 620 fibre-to-dust ratios obtained from the parallel measurements, it was shown that fibre-to-dust ratios in the mine differed by season (higher ratios in winter than in summer) and in the factories varied by factory in the a priori expected direction of ratios increasing with the stages of the asbestos-enrichment process. Furthermore, the fibre-to-dust ratio depended on the measured dust concentration, and the ratios had to be extrapolated to factories without parallel measurement data and to earlier years. Overall, the median fibre to dust ratios in the factories were 1.15, 0.98, and 0.66 in 1995, 2007, and 2013–2014 respectively. Corresponding figures for the mine were 0.92 and 1.20 in 2007 and 2013–2014.

### Strengths and limitations

This is the first large-scale international cohort study of chrysotile produced in the Russian Federation and based on the workforce of the largest active chrysotile mine in the world.

Strengths include (1) the large size and relatively long follow-up of the cohort, resulting in a sizeable number of observed deaths; (2) the large female workforce, enabling analyses of female-specific cancer risk and also potentially offering greater insights into the chrysotile dose–response relationship for tobacco-related cancers, given the relatively low smoking prevalence in Russian women in the past; (3) estimation of exposure metrics based on occupational histories, which were almost entirely covered by measurements of dust concentrations (87% of the exposed working years in the factories and 76% in the mine); (4) the exposure assessment and assignment were done independently from the cancer mortality follow-up so that the exposure and outcome data were only combined at the stage of risk analyses; (5) the cohort enrolment, exposure assessment, and vital status follow-up were based on existing databases earlier constructed for study-independent purposes; (6) rigorous data quality checks as described above were implemented throughout the whole data collection process; (7) because no personal contact was required, there was no participation bias in the study; and (8) access to the original text information from the death certificates as recorded by the ZAGS avoiding inconsistencies introduced by using different coders and different medical classifications over time; this helped in particular to identify the 10 deaths from mesothelioma. The study’s findings will have implications for cancer prevention, cancer services planning, workers’ compensation for occupational diseases, and estimation of the cancer burden due to the effects of chrysotile exposure worldwide. Table 4 shows characteristics of other cohort studies of chrysotile workers in mines or processing facilities in comparison to the present Asbest Chrysotile Cohort Study, further illustrating the uniqueness in terms of size and being the first cohort study of miners and millers for which cancer risk can be investigated in female workers [24–28].

**Table 4:** Characteristics of cohorts of chrysotile workers in mines or processing facilities in comparison to the present Asbest Chrysotile Cohort.

Study	Cohort size, total	Cohort size, women only	Start year	Follow-up period	Deaths, all causes	Deaths, lung cancer	Deaths, mesothelioma
	N	N	Year	Years	N	N	N
*Asbest*, *the Russian Federation*							
*Asbest Chrysotile Cohort*	*35*,*837*	*13*,*260*	*1930*	*1976–2015*	*12*,*728*	*679*	*10*
Quebec, Canada [24, 25]							
Chrysotile production industry	9,780	0	1904	1904–1992	8,009	657	38
Balangero, Italy [26]							
Mine and mills	974	0	1917	1946–2013	499	41	8 pleural, 2 peritoneal cancers
Qinghai Province, China [27, 28]							
Mine and mills	1,539	0	1958	1981–2006	428	56	-

Some weaknesses are inherent in the design of a historical cohort study including (1) spanning decades including time periods before electronic data records, (2) that the advantage of being based on existing databases comes with the disadvantage that those were designed and maintained for an original purpose other than epidemiological research, and (3) not having direct contact with cohort members to collect information on potential confounders. Although there is a wealth of information on dust concentrations, fibre concentrations had to be estimated from rather contemporary parallel measurements of dust and fibre concentrations in 1995, 2007 and 2013–2014. This introduces uncertainty in the extrapolation of fibre concentrations to the earlier time periods and especially at higher dust concentrations (>15 mg/m^3^). Exposure misclassification, i.e. underestimation of exposure, is also inherent when workers have worked elsewhere before or after their employment in the eligible enterprises of JSC UralAsbest. In Asbest however, an industrial mono-town, there were limited employment possibilities with exposure potential, but some workers may have worked in mines or enrichment factories in other parts of Sverdlovsk oblast or the Russian Federation. Although not contributing to the occupational exposure, there was some background exposure to dust and fibres especially in the past with its high mining activities, given the close proximity of the town of Asbest to the mine (Fig 1a) and the old (now destroyed) enrichment factories [29, 30].

Having no individual smoking data is a weakness when analysing tobacco-related cancers [24, 31]. About 12% of the cohort were censored before 2015, the vast majority because of moving away from the oblast, which may impact on the risk estimation if this group was highly selective; but we did not find any strong evidence for this. Finally, the study outcome is cancer mortality, rather than cancer incidence, but notably many of the cancers of interest have poor prognosis. It should also be noted that the average age of death of cohort members is relatively young, reflecting the life expectancy of the Russian Federation in general. Asbestos-related cancers occur rather at higher ages, so that competing causes of deaths, especially the large number due to external causes of death, may have an impact on the results.

### Perspectives

In summary, the Asbest Chrysotile Cohort Study is unique in many ways. This retrospective cohort study of the workforce of the world’s largest operating chrysotile mine in the town of Asbest, Sverdlovsk oblast, the Russian Federation, includes 35,837 workers, of which 13,374 are women. Cumulative exposures to dust (mg/m^3^-years) could be estimated for each worker based on detailed occupational histories extracted from company’s archives and linked to more than 90,000 measurements of dust in the factories since 1951 and in the mine since 1964. Cumulative exposure to asbestos fibres (fibres/cm^3^ years) were based on cumulative dust estimates and conversion factors derived from three series of parallel dust/fibre measurements (n = 620) in the years 1995, 2007, and 2013–2014. Each worker is followed up for cause-specific mortality, derived from official death certificates.

As main limitation however, the high prevalence of smoking as well as the large number of deaths from circulatory system diseases, and external causes and the relatively low life expectancy (59.4 years in men and 66.5 years in women), may impact on the analyses of the chrysotile-related cancer mortality. Notably, this is a limitation reflecting the real-life situation and not a limitation of the study design.

Some of the weaknesses can be overcome if a prospective follow-up of active workers and retired workers still living in Asbest, i.e. ~ 50% of the current cohort, would be initiated. Other disease risk factors could be assessed by questionnaire and through annual medical examinations. Biological samples could be taken to investigate susceptibility, gene–environment interactions, or early detection markers of chrysotile-related disease. Active follow-up of the Asbest Chrysotile Cohort could include cancer incidence and non-cancer outcomes having low mortality.
